# Design and simulation for seeding performance of high-speed inclined corn metering device based on discrete element method (DEM)

**DOI:** 10.1038/s41598-022-23993-1

**Published:** 2022-11-12

**Authors:** Guo Jun, Yang Yue, Muhammad Sohail Memon, Tan Chuang, Wang Linyu, Tang Pei

**Affiliations:** 1 School of Automotive Engineering, Yancheng Institute of Technology, Yancheng, 224051 Jiangsu China; 2 Faculty of Agricultural Engineering, Sindh Agriculture University, Tandojam, 70050 Pakistan

**Keywords:** Mechanical engineering, Software

## Abstract

Mechanical precision corn seed-metering planter has a compact structure, missed and repeated seeding advantages during high-speed operation. In this regard, the current research study focuses on the development of a corn seed planter that features an inclined seed-metering device. The spatial layout of the seed-metering device is optimized to change the seed-filling mode to meet the needs of high-speed operation. Firstly, the mechanical characteristics and seeds in the metering device chamber were analyzed, and then the seed-filling stress model was established. Secondly, a mechanical model for corn seed particles was developed for virtual simulation tests and numerical analysis using the discrete element method (DEM) and EDEM software. Moreover, a quadratic rotating orthogonal center combination test was implemented by setting the inclination angle of seed-metering device *θ*(*A*)*,* machine ground speed *v*(*B*)*,* and rotation speed of metering disc *n*(*C*) as the influence factors, with the missed seeding rate M and the seed-filling stress S as the evaluation indices. The results indicated that the most significant factors affecting the missed seeding rate, seed-filling stress, S, were the rotation speed of the metering disc (*n*) > machine ground speed (*v*) > inclination angle of the metering disc (*θ*) and inclination angle of the metering device (*θ*) > rotation speed of the metering disc (n) > machine ground speed (*v*), respectively. However, the field verification test shows that the optimized corn seed-metering planter achieved mean values of *M* = 4.33, *Q*(qualified seeding rate) = 92.83%, and R(repeated seeding rate) = 2.84%, with average relative errors of 1.17% compared to the simulation tests and the accuracy and effectiveness of the DEM simulation model was verified. Therefore, the developed corn seed-metering device meets the industry standards and operation requirements for precise corn sowing, and technical support can be given for future studies of similar precision seeding equipment.

## Introduction

The development of an appropriate planter is the primary technology involved in corn precision seeding. It comprises a traction device, ditching device, fertilization device, seeding unit, transmission system, soil covering and pressing device, and auxiliary components. During precision seeding operation, the technical requirements include: (i) The traction device is stably controlled with the ground height to ensure that the ditching depth is consistent with seeding depth; (ii) The ditching device can prevent stubble blocking, improve stubble breaking, and reduce soil disturbance; (iii) The seeding unit has good uniformity during high-speed operation; (iv) The soil covering and pressing device make soil covering uniform^1^. Among them, the seed-metering device is the main component of the planter. The corn groups can be separated and quantitatively divided to form a uniform and orderly seed flow, and it was seeded into the soil accurately to control the plant spacing of corn seeds in the field. Its operational performance directly affected seeding quality and efficiency, and the development of precision seed-metering devices for agronomic requirements is vital for the advancement of corn precision seeding technology^2^.

However, the most widely used corn planters in the market are mechanical and pneumatic^3–5^. In the mechanical aspect: metering device of the finger clip and the inclined disc has been developed to overcome the disadvantages of low seeding speed^6^. German scholars conducted theoretical research on the seed-metering device with the inclined disc and analyzed the relationship between the shape of the spoon in the seed-metering disc and seeding accuracy^7^. Another study by^8^ optimized the structure of key components of seed-metering devices according to the shape of various seeds. The Stan-hay company uses flexible seed-guiding belts for seed filling in order to prevent the seed from bouncing and colliding with the soil seedbed during the filling process^9^. The company John Deere developed a brush belt seed-guiding component, which fixed the bouncing and rolling issues when seeds fell into the groove^10^ Also, Li et al.^11^ designed a corn metering device with a centrifugal cone disc, which investigated the various types of seed-filling stress in the device's chamber and resolved the instability issues.

In contrast, Wang et al.^12^ developed a corn metering device that included a finger spoon, and their research was based on the limit-guiding effect of dynamic and fixed finger spoons. Moreover, another research was conducted by Liu et al.^13^, who analyzed the filling stress of corn seeds under different postures and a horizontal seed-metering device with an inclined rectangular hole. Also, Ahmad et al.^14^ and Anantachar et al.^15^ attempt to use computer simulation and artificial neural network models to optimize and predict the seed-metering device's performance with the inclined disc.

In addition, many businesses began to merge hydrodynamics and mechanics to create numerous pneumatic seed-metering devices with the advent of precision seeding technology^16,17^. A blade is added inside the seed-metering device to form a double-sided seed-cleaning^18^, and it can improve the efficiency of single seed sowing. Additionally, Monosem company used a variety of material coupling technology to reform the material of the air-suction seed-metering device^19^. Amazone has also developed a seed-metering device with a fixed front cover and a rotatable suction chamber^16^. To enhance seed-filling efficiency, the Case IH company created a finger metering disc made of flexible materials^20^. Additionally, researchers began to produce positive pressure, air blowing, and central cluster seed-metering devices based on the negative pressure air suction^21–24^. Yan et al.^25^ designed an air-suction seed-metering device in which the seed-metering disc rotates synchronously with the negative pressure chamber. At the same time, vibration and ultrasonic technology have been widely used in the design of the seed-metering device, and it will become the focus in future^26–31^.

Our research team proposes the idea of precision seeding by combining the principles of conservation tillage and the principle of the corn planter with the mechanical and pneumatic seed-metering device. These three ideas come together to form the concept of precision seeding. In response to the urgent problem of the complex structure and low working efficiency of the traditional corn planter, a corn planter with a single-chamber and inclined device suitable for high-speed was developed. The corn planter can complete the multiple operation processes of straw-stubble breaking, seeding and soil covering. Optimization design of the vital structural components was conducted, and experimental analysis of the functional performance and operation effect was conducted to obtain the optimal combination of working parameters when the corn planter exhibits its best performance.

## Materials and methods

### Corn planter structure and working principle

#### Planter structure and technical parameter

**Figure 1:**
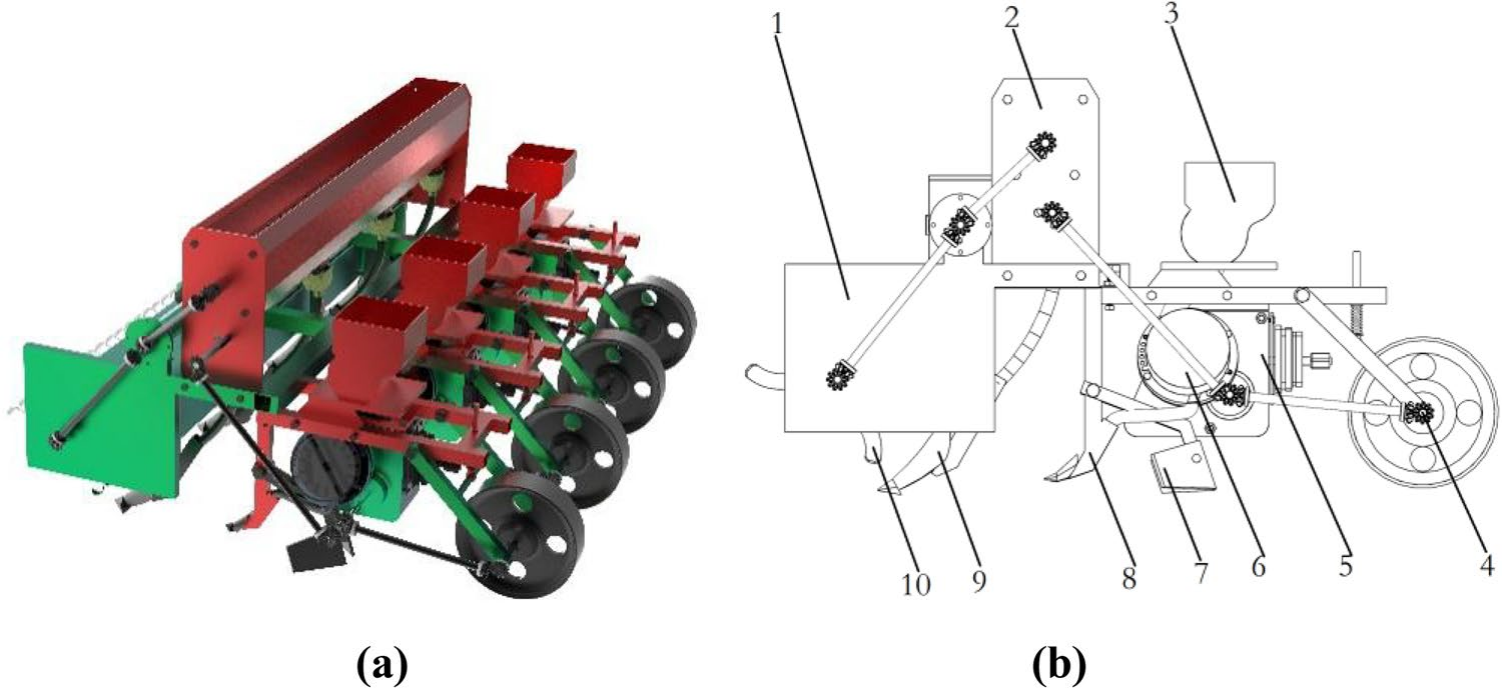
The overall structure of the corn planter with the inclined seed-metering device. (**a**) 3D graphic of corn planter; (**b**) 2D graphic of corn planter.

The main structure of the corn planter includes a seed fertilizer box, fixed frame, ditch openers, inclined seed-metering devices, soil cover devices, rotary blades, walking wheels and power transmission (Fig. 1) and technical parameters are depicted in Table 1.

**Table 1:** Main parameters of the planter with inclined seed-metering device.

Parameter	Value
Dimensions (length × width × height)	1800 × 2070 × 1650
Speed (m/s)	1.4–3.3
Power (hp)	75–100
Number of rows	04
Distance between rows (mm)	500
Acupoint distance (mm)	200
Gear (Nos.)	08

**Figure 2:**
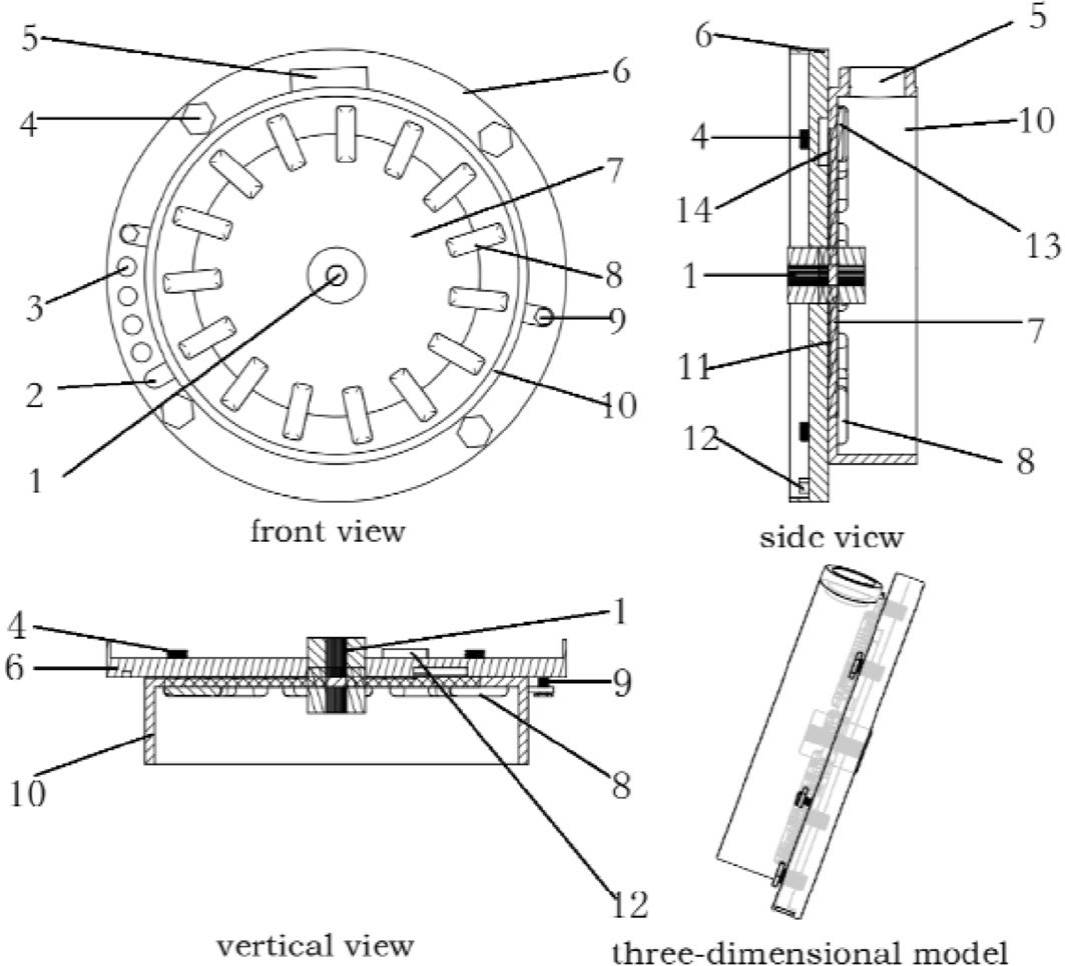
Structure of inclined seed-metering device.

The basic idea of an inclined seed-metering device is to use the seed-metering shell space fully. It is mainly composed of basic disc 6, cover disc 10, seed-metering disc 7, seed-metering spoon 8, seed-blocking 14 and fixing bolts 4 and 9, connecting the basic and cover disc. Moreover, the structure of the inclined seed-metering device is shown in Fig. 2.

#### Working principle of inclined seed-metering device

**Figure 3:**
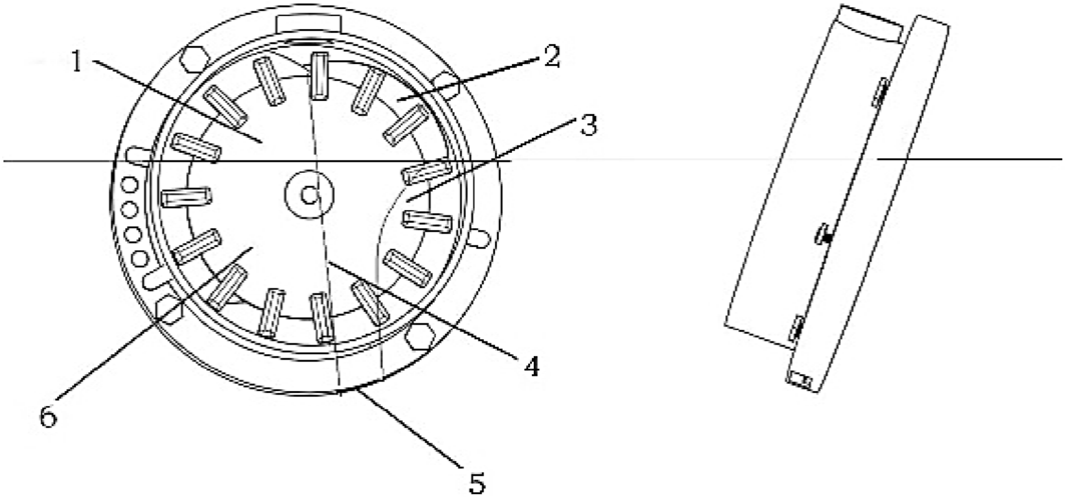
Working zone of the inclined seed-metering device.

As depicted in Fig. 3, the corn seed is subjected to the squeezing stress of the seed clusters, seed gravity stress, and centrifugal stress generated by the rotation of the seed-metering device in the seed-filling area. Under three stresses, the corn seeds enter the spoon and rotate with the seed metering disc. When the seeds in the spoon move to the highest point of the disc and enter the zone of seeds-cleaning, some seeds at the edge of the spoon fall back to the seed-metering chamber under the action of gravity, and only one seed remains in the spoon and continues to move with the rotation of the metering disc until entering the seed protection zone. When the seed reaches the seed-feeding zone, the seed passes through the shell of metering devices under gravity and centrifugal stress. In the meantime, the seed is thrown to the outlet at a certain speed along with the tangent direction of the metering disc, and the process of seed-metering is completed in turn.

#### Mechanism of seed-filling

**Figure 4:**
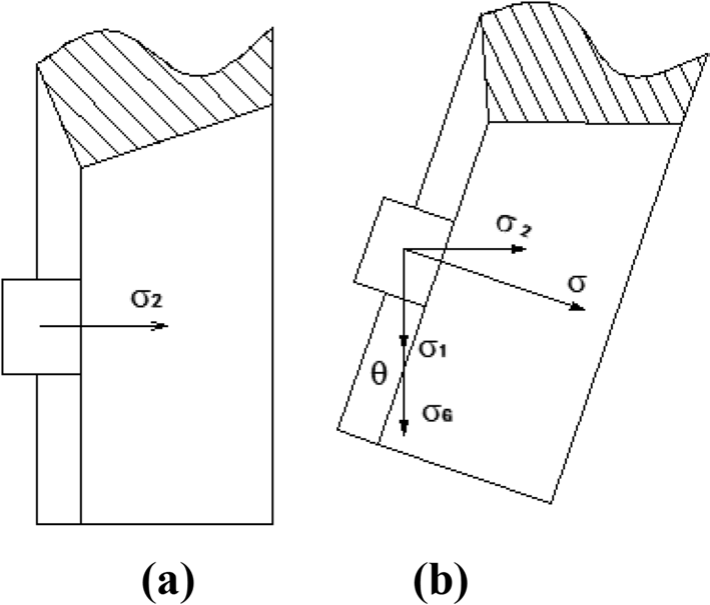
The contrast in seed-filling stress of two kinds of seed-metering devices. (**a**) Vertical seed-metering device; (**b**) inclined seed-metering device.

In order to analyze the seed-filling mechanism of the inclined seed-metering device, we selected a vertical seed-metering device as the contrast. The vertical seed-metering device mainly depends on the horizontal lateral stress (${\sigma }_{2}$) between seed clusters to complete seed-filling, as shown in Fig. 4a. However, the horizontal lateral stress (${\sigma }_{2}$) is limited^28,32^, so when the metering device's operation speed increases, the corn seeds' time to enter the spoon becomes shorter, and the effect and efficiency of seed-filling and efficiency decrease sharply. For the inclined seed-metering device, the seeds in the seed-filling zone of the disc above are subjected to gravity stress ${\sigma }_{G}$, the seeds in the seed-filling zone of the disc lateral are subjected to horizontal lateral stress ${\sigma }_{2}$, the seeds in the seed-filling zone of the disc below are subjected to the combined stress of vertical compressive stress ${\sigma }_{1}$ and gravity stress ${\sigma }_{G}$. During the seed-filling process of the inclined seed-metering device, the horizontal lateral compressive stress of seed clusters ${\sigma }_{2}$, the components of vertical compressive stress ${\sigma }_{1}$ and gravity stress ${\sigma }_{G}$ are used. Figure 4b shows that compared with compressive stress in the vertical seed-metering device, the types of seed-filling stress are increased. Therefore, the seed-filling capacity of the metering device can be significantly improved at high speed. In order to simplify the calculation, the corn seed is simplified into a square model.

#### Model of seed-filling stress

The inclined seed-metering device has three stresses acting on seed-filling: the lateral compressive stress of seed clusters, the vertical compressive stress of seed clusters upper and the gravity stress of seeds. Seed-filling stress of the inclined seed-metering device is obtained by adding three stresses to the vector. According to the theoretical analysis, Seed-filling stress is positively correlated with the inclination angle, but when the seed-metering device is horizontal (i.e.* θ* = 90°), it is easy to damage the seeds and unable to clean excess seeds. Therefore, Seed-filling stress does not have a linear relationship with the inclination angle of the seed-metering device. Relevant literature shows that the lateral stress distribution of particles on the container wall is related to the mechanical properties of particles and stacking depth of particles^33^.

**Figure 5:**
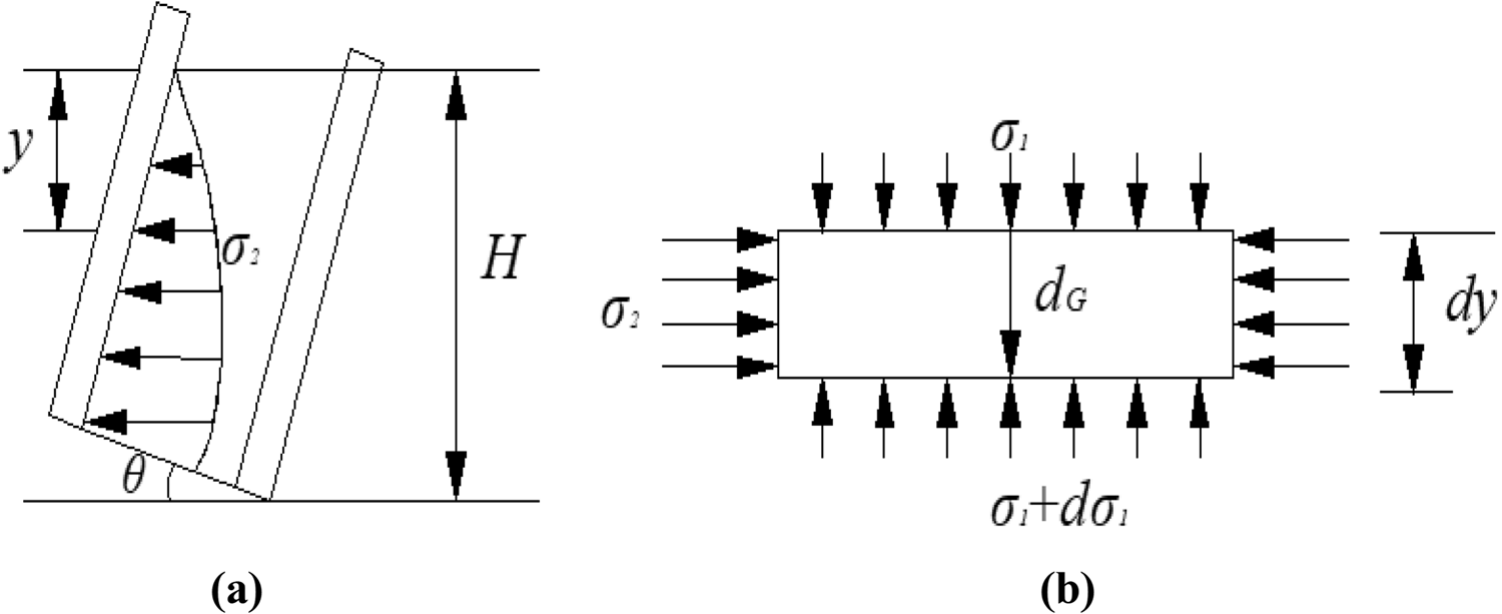
The stress distribution unit of the seed-filling area.

Moreover, another study by Zhou^34^ reported that the seed container of the seed-metering device is simplified to a deep chamber, and it is assumed that the vertical compressive stress on the horizontal plane of the seed chamber is constant. The depth (*y*) of the seed layer from the horizontal plane is taken as the research object, taking out tiny seed layer depth (*dy*). The stress is shown in Fig. 5.

If the cross-sectional area of the seed layer is A and the circumference is C, the force balance equation is:

1 $${\sigma }_{1}A+\gamma Ady-\left({\sigma }_{1}+d{\sigma }_{1}\right)A-{f}_{s}K{\sigma }_{1}Cdy=0$$

After simplification, Eq. (2) is obtained:

2 $$\frac{d{\sigma }_{1}}{dy}=\gamma -\frac{K{f}_{s}{\sigma }_{1}}{{R}_{h}}$$

When *y* = 0, ${\sigma }_{1}$=0, after the integral, Eq. (3) is obtained:

3 $$\left\{\begin{array}{l}{\sigma }_{1}=\frac{\gamma {R}_{h}}{{f}_{s}K}\left[1-{e}^{\frac{-K{f}_{s}y}{{R}_{h}}}\right]\\ {\sigma }_{2}=\frac{\gamma {R}_{h}}{{f}_{s}}\left[1-{e}^{\frac{-K{f}_{s}y}{{R}_{h}}}\right]\end{array}\right.$$

In the formula, *γ* is the weight of the bulk material, the value of corn is 9.02 *kN/m*^*3*^ (the water content is 18%); *R*_*h*_ is the hydraulic radius, the value of the corn is 0.0085 m^35^. *K* is the pressure ratio:

4 $$K=\frac{{\sigma }_{2}}{{\sigma }_{1}}=\frac{1-\mathit{sin}\,\phi }{1+\mathit{sin}\,\phi }$$

In the formula, $\phi$ is the friction angle of the material, the value of corn is 27° (the water content is 18%), and the *K* is 0.35. *f*_*s*_ is the static-sliding friction coefficient between the material and chamber wall, and the value is 0.298^35^, *y* is the variable of seed layer depth, mm.

5 $$y=80\%\cdot d\cdot \mathit{cos}\,\theta $$

In the formula, 80% is the ratio of the seed clusters' height to the seed chamber's overall height; *d* is the diameter of the seed-metering disc, mm, the value is 180 mm; $\theta$ is the inclination angle of the seed-metering device (°).

The seed is filled in the device with the combined stress of horizontal lateral compressive stress, vertical compressive stress and gravity stress between seed clusters, and the filling stress is:

6 $$\sigma ={\sigma }_{G}\mathit{sin}\,\theta +{\sigma }_{1}\mathit{sin}\,\theta +{\sigma }_{2}{\text{cos}}\,\theta $$

where ${\sigma }_{G}$ is the gravity stress of the seed, ${\sigma }_{G}=G/ab$, the weight of 100 corns is used in the experiment. The value is 30 g; *a, b* is the length and height of the corn seed, the value is 12 mm and 11 mm, respectively.

Formula (3) and formula (5) are substituted into formula (6), and formulas (7), (8), (9) are obtained:

7 $${\sigma }_{1} \, \left(\theta \right)=0.74 \left(1-{\text{e}}^{-2.45\text{cos}\,\theta }\right) $$

8 $${\sigma }_{2} \, \left(\theta \right)=0.26 (1-{\text{e}}^{-2.45\text{cos}\,\theta })$$

9 $$\sigma \left(\theta \right)=0.23\mathit{sin} \, \theta +0.74\left(1-{\text{e}}^{-2.45\text{cos}\,\theta }\right)\mathit{sin}\,\theta +0.26 (1-{\text{e}}^{-2.45\text{cos}\,\theta })\text{cos}\,\theta $$

**Figure 6:**
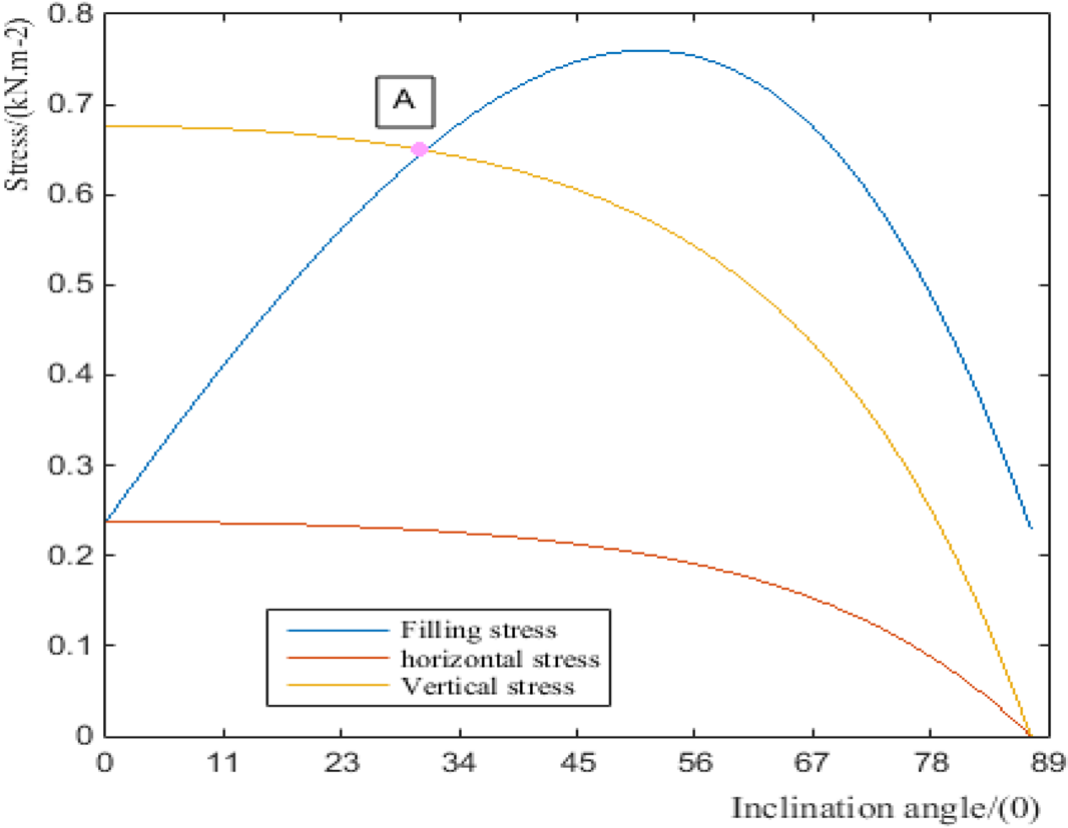
Relational graph about stress with the inclination angle of the metering device.

The relationship between the composite filling stress and the inclination angle of the seed-metering device is calculated by MATLAB, as shown in Fig. 6. When the inclination angle *θ* is 45° to 55°, the seed-filling stress is the largest, and the value of maximum stress is about 0.77 kPa*,* but at point A, the vertical stress decreases sharply. Thereby, the best inclination angle of the metering device is 15° to 30°.

### Simulation model creation and parameter setting

**Figure 7:**
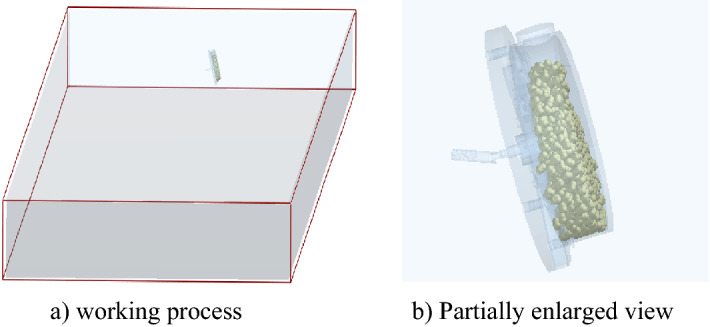
Simulation model and simulation process.

**Figure 8:**
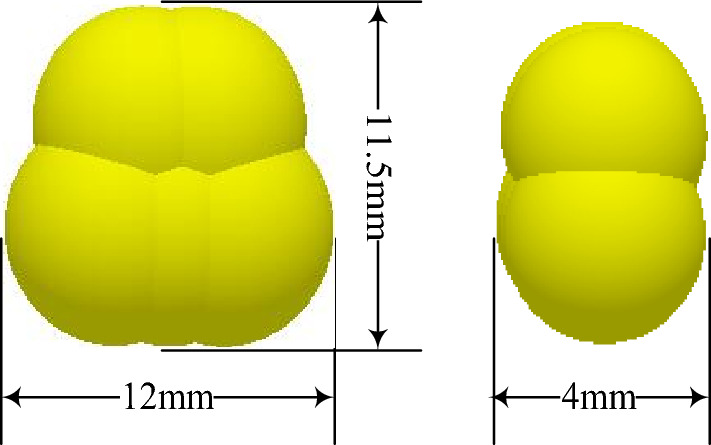
Showing realistic irregularity of single corn seed particle.

The EDM is widely used for the interaction between discrete units and mechanisms of agricultural machinery. The simplified model of the corn planter was imported into the software (EDEM 2018) pre-processing module (Fig. 7). Multi-spherical aggregation was selected to describe the corn seeds' motion feature and filling process. A total of five soft sphere models with diameters of 2.5 and 3 mm, were connected to construct the corn seed particle model for irregular shape and we collected different corn seeds, measured the physical parameters of standard corn seeds, and averaged the parameters of length, width and height, thereby, its shape is modeled (Fig. 8), while the designing process of the corn seeds model is referred to Shi et al.^36^. In this paper, we choose to simulate using a single metering device so that we may save some time.

Table 2 presents corn seed particles' physical and mechanical contact properties determined by random sampling^37^. Considering the number of seeds stored in the metering disc and operating conditions, the number of corn particles was 1000. The rotational speed of the metering disc was in the range of 6–14 rad/s, the inclination angle of the metering device was 15°–30°, the working forward speed of the planter was 1.4–3.3 m/s, and the fixed time step was set to 20% of the Rayleigh time step (6.23 × 10^−5^ s). Whereas to ensure the integrity of corn seed particle movement during simulation, a single simulation was run for the 50 s, and only the test results from the 40 s in the regular working interval were extracted for subsequent statistical analysis^36^.

**Table 2:** Physical and contact mechanical properties parameters.

Parameters	Corn/particle	Cover and basic disc of device/plastic	Metering disc of device/steel
Poisson’s ratio	0.4	0.5	0.3
Shear modulus (MPa)	177	137	7 × 104
Density (kg m^−3^)	1180	1197	7.8 × 103
Contact mechanical parameters	Elastic recovery coefficient	Particle to particle	0.182
		Particle to plastic	0.621
		Particle to steel	0.6
	Rolling friction coefficient	Particle to particle	0.0782
		Particle to plastic	0.0931
		Particle to steel	0.01
	Static friction coefficient	Particle to particle	0.431
		Particle to plastic	0.482
		Particle to steel	0.3
Length, width and height of particle/mm	12 × 4 × 11.5		

### Scheme and method of simulation test

Necessary operating motion parameters based on actual working conditions for the imported discrete Model of the one inclined device were set. After that, simulations were conducted in the virtually generated corn particles, according to the operational specifications and requirements provided in Agriculture Industrial Standard NY/T3491-2002 Applicability evaluation method of corn no-tillage machine^38^.

According to the theoretical analysis of seed-filling in the process of corn seeding by Liu et al.^39,40^. and Chen et al.^41,42^. the main working parameters affecting the seed motion process and seed-filling in the disc, namely the metering disc's rotary speed *n*, the metering device's inclination angle *θ*, and working forward speed *v*, were selected as the test factors. Furthermore, missed seeding rate *M* and seed-filling stress *S* were set as evaluation indexes to characterize the seeding quality. Virtual tests on the quadratic rotating orthogonal center combination with three factors and five levels were carried out to evaluate the operating performance of the planter with seed motion and seed-filling process. Similar research by Shi et al.^36^; He et al.^43^ and Cay et al.^44^. indicated that the planter's ground speed is 1.4–3.3 m/s, and the metering device's inclination angle is 15°–30°, whereas the rotary speed of the metering disc is 6–14 rad/s. Therefore, based on actual practical experience, the appropriate levels of the test factors were established, as indicated in Table 3, and the 20 groups of testing schemes are depicted in Table 4.

**Table 3:** Factors and levels of a virtual test.

Test factors		Coded value					Interval Δ_*i*_
		(−$\gamma$)	(−1)	0	(1)	($\gamma$)	
*A*	Inclination angle of the metering device (°)	15.95	18	21	24	26.05	3
*B*	Working forward speed (m/s)	1.73	2	2.4	2.8	3.07	0.4
*C*	Rotational speed of the metering disc (rad/s)	6.636	8	10	12	13.364	2

**Table 4:** ANOVA of regression model.

Indicator	Source of variance	Sum of squares	Freedom	Mean square	F	P	Significant
***M***	**Model**	26.71	9	2.97	6.99	0.0027	**
	A	0.53	1	0.53	1.24	0.2914	
	B	0.66	1	0.66	1.55	0.2412	
	C	7.39	1	7.39	17.41	0.0019	**
	AB	1.13	1	1.13	2.65	0.1346	
	AC	6.13	1	6.13	14.43	0.0035	**
	BC	1.13	1	1.13	2.65	0.1346	
	A^2^	2.25	1	2.25	5.29	0.0443	*
	B^2^	0.68	1	0.68	1.61	0.2329	
	C^2^	8.07	1	8.07	19.01	0.0014	**
	Residual	4.24	10	0.42			
	Lack of fit	2.24	5	0.45	1.12	0.4511	
	Pure error	2.00	5	0.40			
	Total Corrected	30.95	19				
***S***	**Model**	0.13	9	0.014	7.71	0.0018	**
	A	0.021	1	0.021	11.18	0.0074	**
	B	0.00033	1	0.00033	0.18	0.6805	
	C	0.0062	1	0.0062	3.37	0.0962	
	AB	0.068	1	0.068	37.14	0.0001	**
	AC	0.0098	1	0.0098	5.32	0.0438	*
	BC	0.0008	1	0.0008	0.43	0.5249	
	A^2^	0.021	1	0.021	11.42	0.0070	**
	B^2^	0.00008	1	0.00008	0.046	0.8339	
	C^2^	0.00012	1	0.00012	0.064	0.8049	
	Residual	0.018	10	0.0018			
	Lack of fit	0.011	5	0.0021	1.38	0.3667	
	Pure error	0.0078	5	0.0016			
	Total corrected	0.15	19				

**Figure 9:**
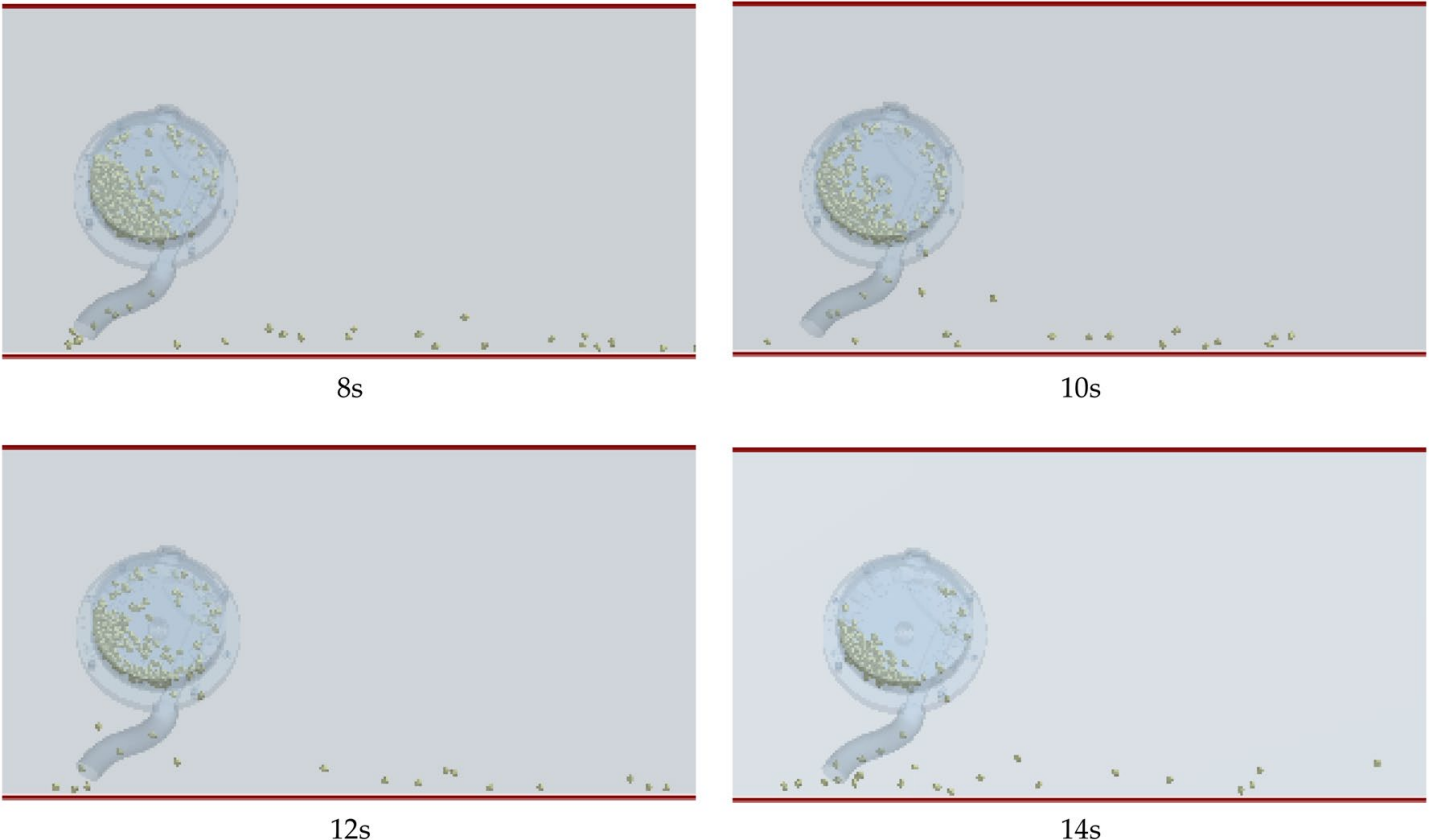
Variable seed motility behavior at various times.

**Figure 10:**
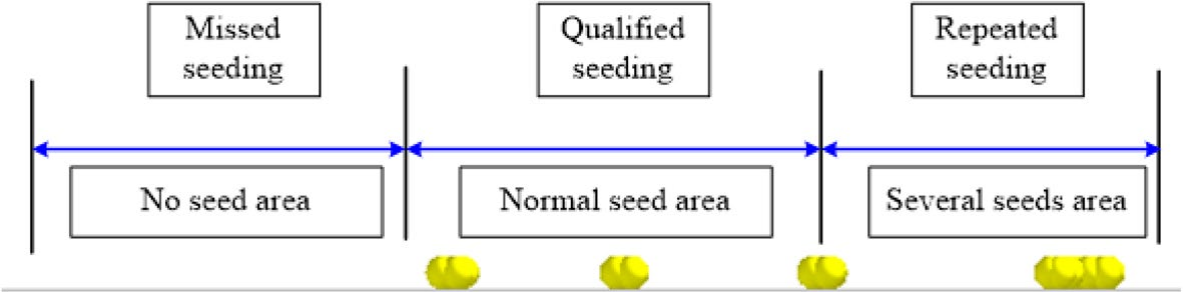
Distribution of corn seeds in the forward direction after simulation.

Note: By calibrating 20 m in the forward direction of the planter and marking at 0.2 m intervals, 100 marking points are obtained, and finally the seeds distribution on 100 marking points are counted. If there is no seed, it is recorded as seed missing.

During the tests, the planter was adjusted with different operation parameters (metering device's inclination angle, working forward speed, and metering disc's rotary speed). After waiting for its calibration and stable condition to be reached, the planter passed through the virtual test area to ensure test accuracy in the determination region. After that, missed seeding rate (*M*) and seed-filling stress (*S*) were measured to study the effects of the operation parameters, the movement behavior of the seed at different times, shown in Fig. 9, and the distribution of seeds after the simulation is shown in Fig. 10. Following the completion of every single test, 300 points are ranked in the length direction of the virtual operation area (the length is 60 m), 100 points in a group (the length is 20 m), and the number of points (no seed, single seed and multiple seeds) was counted successively by the post-processing module of the EDEM software. The resulting values of the three groups were averaged. The corresponding test evaluation indices of the missed seeding rate(*M*) were calculated by the following equation:

10 $$M=\frac{n}{N}\times 100\%$$

where, *M* is the missed seeding rate*; n* is the number of collection points seedless; *N* is the number of theoretical collection points (100) ,because the length is 20 m, and the acupoint distance is 0.2 m.

### Field test conditions and method

The field experiment under no-tillage seeding was conducted at the *Yandong* community farm of Yancheng from June to July 2021. The test area was approximately 2.5 *hm*^*2*^, the previous crop was wheat, the soil type was sticky, the average grass-grain ratio was 1.5, and the average grass-grain mass was approximately 2.4 kg/m^2^. According to Chinese Agriculture Rotary tillage machinery standard^45^**,** five points are sampled shall by diagonal method, taking the sample from each point in the depth of 0–5, 5–10 and 10–15 cm with a soil box, and the sampling amount of soil from each layer is more than 30 g (removed impurities such as stones and straw residuals). After that, the drying method obtains the average moisture content of soil from each layer. The values are 28.4%, 29.7% and 31.2%, respectively. Meanwhile, the average cone index of soil from each layer was measured as 50.33, 125.15 and 289.47 kPa by soil firmness meter.

**Figure 11:**
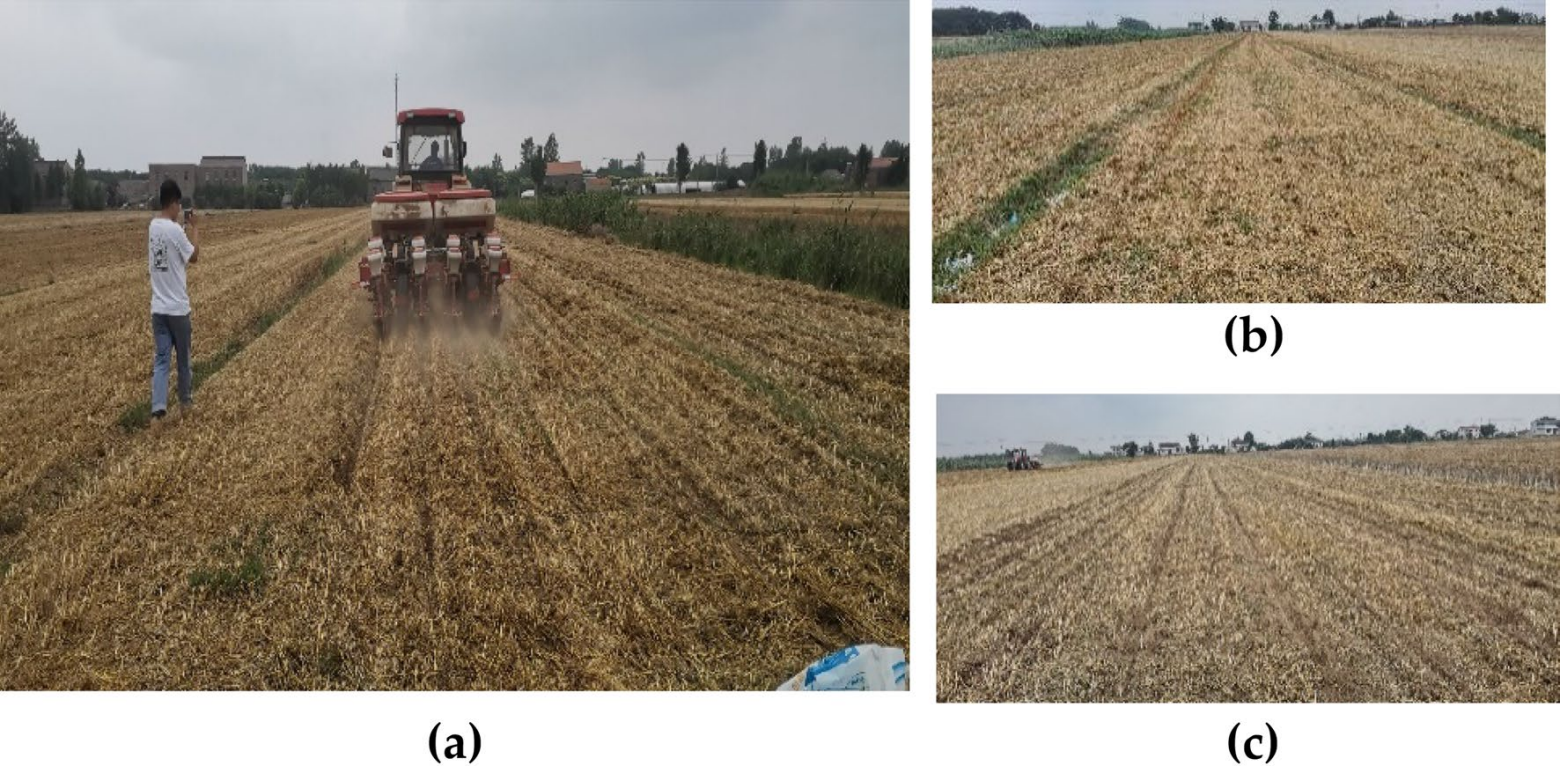
Field experiment under inclined seed-metering. (**a**) In-situ operation of the inclined seed-metering device; (**b**) field view before operation; (**c**) field condition after operation of the metering device.

YITUO DFH1204 hosted a seeding machine-wheeled tractor with a three-point suspension. The test process was carried out under the performance requirements provided in the Chinese Agriculture Single Grain (corn or soybean) No-Till Planter^46^. According to the response surface optimization experiment, the optimal parameter combination is obtained: metering disc's rotary speed *n*′ = 10.5 rad/s, working forward speed *v* = 2.73 m/s, and metering disc's inclination angle *θ* = 24°. The data acquisition method is the same as in “Interactive analysis and discussion”. The number of corn seeds in each collection point was counted in the effective working area, the tests were repeated three times for each group, and the mean values were obtained. A total of six groups of tests were carried out, and the length of each test was 60 m, as shown in Fig. 11.

## Results and discussion

**Figure 12:**
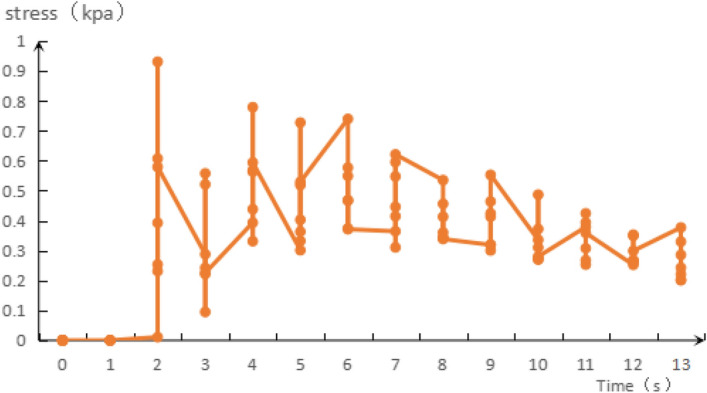
Stress changes at different times.

The simulation test results are based on the design scheme presented in Table 4, and one group of stress changes is shown in Fig. 12, including 14 analysis factors and 06 zero-point tests for estimating the error. Quadratic multiple regression analysis of the results in Table 4 was performed using the Design-Expert 10.0 software. While the regression models between the influencing factors and evaluation indices were established as follows:

11 $$\left\{\begin{array}{l}M=4.02+0.20A+0.22B-0.74C+0.38AB+0.88AC-\\ 0.37BC+0.39{A}^{2}+0.22{B}^{2}+0.75{C}^{2}\\ S=0.62+0.039A+0.0049B-0.021C+0.092AB+0.035AC\\ -0.01BC+0.038{A}^{2}-0.0024{B}^{2}+0.0028{C}^{2}\end{array}\right.$$

### Variance analysis and discussion

The F-test and analysis of variance (ANOVA) were performed on the regression coefficients in the regression models of the evaluation indices *M* and *S*, and the results are presented in Table 4. According to the *P* values of model and the lack of fitting(*P*_*LM*_ = 0.4511 > 0.05 and *P*_*LS*_ = 0.3667 > 0.05; *P*_*MM*_ = 0.0027 < 0.01 and *P*_*MS*_ = 0.0018 < 0.01), *P*_*LM*_ and *P*_*LS*_ were not significant, *P*_*MM*_ and *P*_*MS*_ were significant, indicating that the abnormal error is very small between the obtained equation and the actual fitting, and the regression equation is valid.

It can be seen from Table 4 that the three factors of inclination angle, forward speed and rotational speed have different effects on missed seeding and filling stress. According to the *P* values of C, AC and C^2^, they had highly significant influences on missed seeding rate; According to the *P* values of A, AB, and A^2^, they had highly significant influences on filling stress. Therefore, the primary and secondary orders for the influences of each test factor on the missed seeding rate *M* and filling stress *S* were rotational speed of the metering disc (*n*) > working forward speed (*v*) > Inclination angle of the metering device (*θ*), and inclination angle of the metering device (*θ*) > rotational speed of the metering disc (*n*) > working forward speed (*v*), respectively.

### Interactive analysis and discussion

**Figure 13:**
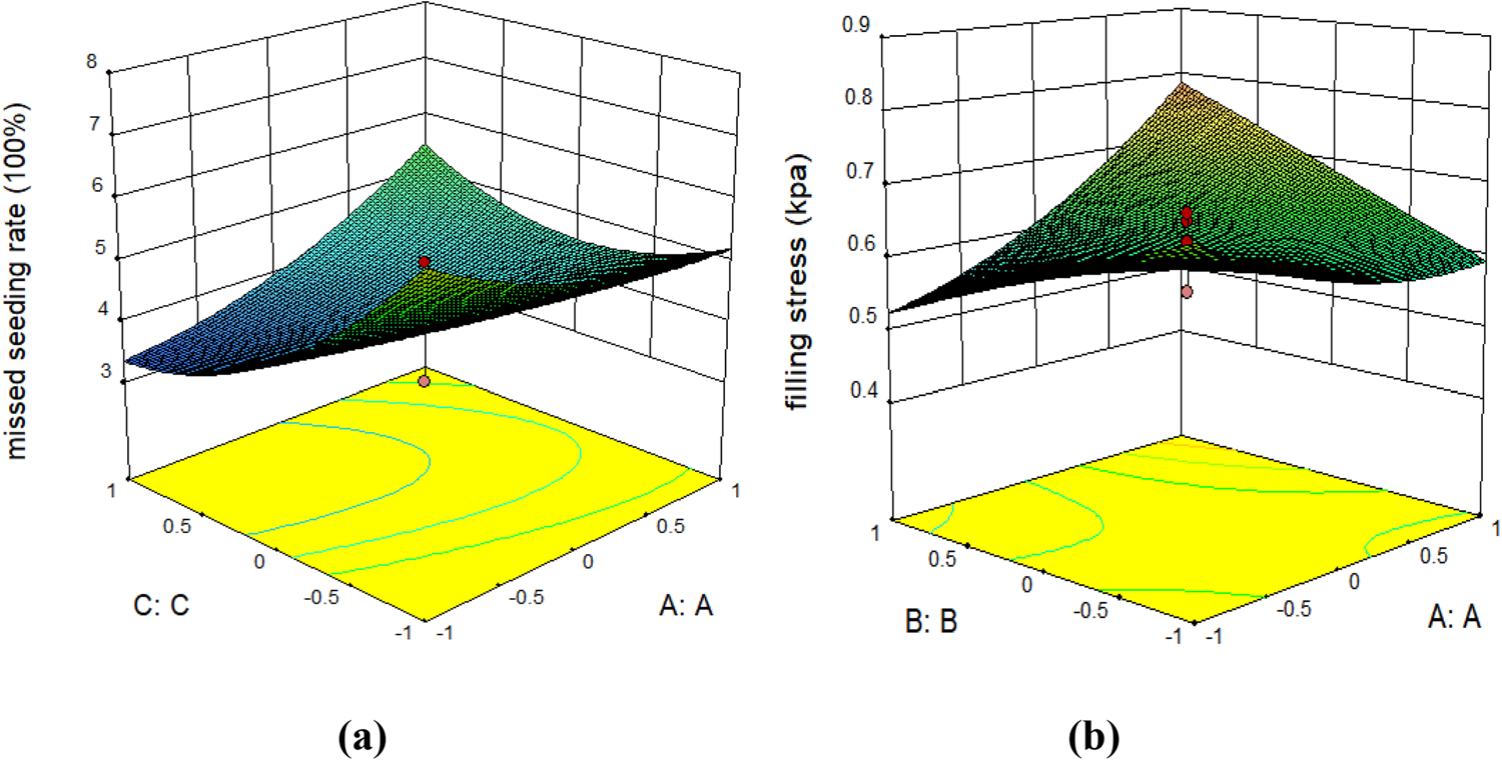
Effect of interaction between factors on missed seeding rate and coefficient of filling stress. (**a**) Missed seeding rate; (**b**) seed-filling stress.

In order to describe the influence of interactive factors on missed seeding rate and filling stress clearly, dimension reduction is performed in current paper^36^, and the insignificant items are removed(AB and BC in index *M* and BC and AC in index *S*). Therefore, the corresponding response surfaces were generated, as shown in Fig. 13.

According to Fig. 12, the maximum stress in the simulation analysis is 0.93 kPa, and the maximum stress in the theoretical calculation is 0.77 kPa. The maximum values of the two are close. In addition, when the time is 4–6 s, the value fluctuates around 0.6kPa, and the maximum seed-filling stress is 0.78 kPa. When the time is more than 7 s, the value fluctuates between 0.3 and 0.4 kPa, and the maximum seed-filling stress is 0.6 kPa. The overall change trend shows that it increases first and then decreases, which is consistent with the theoretical change trend of the value calculated.

Moreover, according to the response surface analysis for the missed seeding rate *M* (Fig. 13a), the inclination angle of the metering device (*θ*) was constant. Furthermore, the missed seeding rate *M* decreased with the increase in the rotational speed of the metering disc (*n*), but to a specific value, following which *M* tended to increase gradually, and when *n* was within the range of 10–12 rad/s, the minimum value of *M* appeared. This was due to the metering disc's rotating speed, which enhanced the fluidity of the seeds in the cavity and allowed for more uniform filling of the seed spon. Under the shunting action of the guide device at the seed-metering port, the seeds are evenly discharged, and the missed seeding rate is decreased. However, when *n* increased, turbulent flow of the seeds in the chamber occurred under the higher centrifugal inertial stress, following which parts of the seeds were pressed against each other and clustered together, leading to an increase in the missed seeding rate. As the forward machine speed *v* increased, the residence time of seeds near the seed-metering port of the guide device was reduced, which led to most seeds returning to the chamber, and the missed seeding rate would gradually increase. Further analysis demonstrated that the response surface for *M* changed more rapidly in the direction of the rotational speed of the metering disc than forwarding speed, indicating the rotary speed *n* had a more significant influence on the missed seeding rate *M* than the machine forward speed *v*.

Importantly, in this case, the Inclination angle of the metering device *θ* had no significant effect on *M*. However, as can be observed from the response surface analysis for the coefficient of seed-filling stress *S* (Fig. 13b), under the condition that the machine forward speed *v* remained constant, along with the increase in the Inclination angle of the metering device *θ*, the seed-filling stress *S* became increasingly higher, with the increase of the inclination angle of metering device and the number of seeds, the vertical compressive stress and horizontal compressive stress increased gradually. Nevertheless, once *θ* reached a specific value, the coefficient of seed-filling stress *S* also tended to become stable. Due to the existence of centrifugal stress caused by rotation, when the inclination angle of the seed metering device is 26°, the filling stress *S* reaches the maximum. Meanwhile, if the inclination angle is 90° and there is no centrifugal stress, filling stress *S* is the gravity stress of the seed. When the rotary speed of metering disc *n* was gradually increased, the coefficient of seed-filling stress *S* exhibited an evident trend decreasing because the direction of centrifugal stress caused by rotation is opposite to that of compressive stress, which reduces the filling stress. When the rotary speed *n* was gradually increased, the filling time of seeds in the chamber was shorter, and it was difficult for seeds to enter the seed spoon, so the filling stress decreased sharply. Similarly, further analysis demonstrated that the response surface for *S* in the direction of changing inclination angle *θ* was superior to that in the rotary speed and forward speed direction, indicating that the influence of the inclination angle *θ* was more significant in this interaction. Therefore, *θ* was the main factor affecting the coefficient of seed-filling stress *S*, and the forward speed *v* was non-significant for *S* in this case.

### Comprehensive optimal design

A parametric mathematical model is established by using the maximum filling force (S) and the minimum missing seeding(M) rate as the final optimization objectives. The optimization solution is carried out with the boundary conditions of various factors. The objective function and constraint conditions were established in Eq. (12).

12 $$\left\{\begin{array}{l}\left\{\begin{array}{l}\mathit{max}f(A,B,C)=F\\ s.t.g(A,B,C)=M\le 5\%\end{array}\right.\\ \left\{\begin{array}{l}15.95\le A\le 26.05\\ 0.66\le B\le 1.34\\ 6.636\le C\le 13.364\end{array}\right.\end{array}\right.$$

In addition, the Design-Expert software was applied to optimize and solve the above mathematical Model. The optimal combination of working parameters affecting the missed seeding rate *M* and seed-filling stress *S* of the planter were obtained as follows: inclination angle of metering device *θ* = 24°, forward working speed *v* = 2.73 m/s, and rotary speed of metering disc *n* = 10.5 rad/s, missed seeding rate = 5.25% and seed-filling stress = 0.77kPa*.*

### Comparisons with field measurements

**Figure 14:**
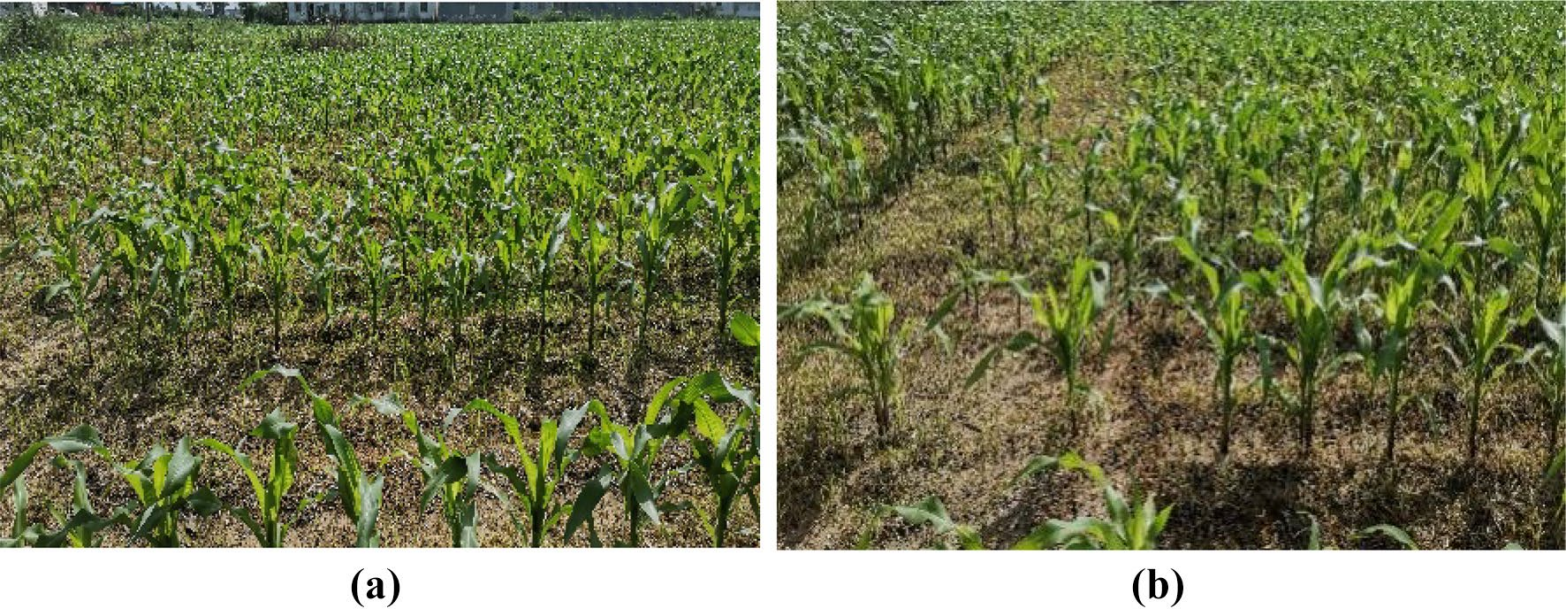
Field validation test and seeding effect under field conditions. (**a**) Inclined seed-metering device; (**b**) vertical seed-metering device.

Combined with the actual field experiment to verify the accuracy of the theoretical derivation and simulation model, Fig. 12 illustrates the field test and working effect. While it is difficult to obtain the filling stress in the field experiment, to compare with the simulation experiment, we use the qualified seeding rate to characterize the filling stress, and the calculation method of the qualified seeding rate is the same as the formula (9). At the same time, field tests of the planter with the inclined seed-metering device and the vertical seed-metering device are compared, as shown in Fig. 14.

The verification test results are summarized in Table 5. When comparing and analyzing the test data in Table 5, it can be observed that the developed planter with qualified seeding exhibited superior performance when using the optimized combination of operating parameters. The average relative errors of the missed seeding rate *M* between the field and simulation test results were only 1.17%, indicating that the established DEM simulation model and virtual test analysis provided certain accuracy and effectiveness. Further analysis revealed that the evaluation index of the repeated seeding rate *R* had a maximum value of 5%, a minimum value of 1%, and a mean value of 2.84%. Moreover, the qualified seeding rate *Q* had a maximum value of 96%, a minimum of 90%, and a mean value of 92.83%. These investigations demonstrate that the optimized combination of operating parameters for the planter with qualified seeding offers rationality and feasibility, and the operation quality can meet the specification requirements of relevant industry standards for the planter.

**Table 5:** Results and comparison of field validation test.

Test	Missed seeding rate *Q*/100%		Relative error /100%	Qualified seeding/100%	Repeated seeding/100%
	Simulation value	Test value			
1	5.25	2	3.25	96	2
2		4	1.25	94	2
3		6	0.75	93	1
4		5	0.25	92	3
5		4	1.25	92	4
6		5	0.25	90	5
Average		4.33	1.17	92.83	2.84

## Conclusions

In this study, a new seed planter with inclined devices was developed. Through the theoretical calculation of the seed-filling stress in the metering device, the seeding process is simulated by using the method of analysis of variance and discrete element software, and finally, it was verified by field experiment. The following conclusions can be drawn.

(i) From the mechanics perspective, the current research study improves the seed-filling capacity of mechanical seed-metering devices to meet the needs of high-speed operation; analyzes the mechanical characteristics and laws of seeds in all directions in the chamber of the seed-metering device. This establishes the Model of seed-filling stress, finds an effective way to improve the seed-filling capacity, and innovatively designs a corn seed metering device with an inclination angle of 15–30 degrees to increase the types of seed-filling stress.
(ii) The primary and secondary factors that affect the missed seeding rate, *M*, and seed-filling stress, *S*, of the planter with inclination angle were rotational speed of the metering disc (*n*) > working forward speed (*v*) > Inclination angle of the metering device (*θ*), and inclination angle of the metering device (*θ*) > rotational speed of the metering disc (*n*) > working forward speed (*v*), respectively. Moreover, the optimal operating parameter combination of the planter was: *θ* = 24°, *v* = 2.73 m/s, and *n* = 10.5 rad/s, which yielded *M* = 5.25% and *S* = 0.77 kPa.
(iii) The field verification test results demonstrated that the average missed seeding rate, *M*, was 4.33%, the qualified seeding rate, *Q*, was 92.83%, and the repeated seeding rate, *R,* was2.84% under the optimized operation parameter combination. The mean relative errors of the simulated test values in missed seeding rate was 5.25%, indicating the accuracy and effectiveness of the established DEM simulation model and virtual test analysis (Supplementary Information).

## Supplementary Information

Supplementary Information.

## Data Availability

All data are presented in this article in the form of figures, tables and supplementary.
