# Supplementary material for: Design and simulation for seeding performance of high-speed inclined corn metering device based on discrete element method (DEM)
Source: Sci Rep. 2022 Nov 12;12:19415. doi: 10.1038/s41598-022-23993-1 (PMC9653446; doi:10.1038/s41598-022-23993-1)
Supplement: Supplementary file 1 — Supplementary Information. [file 41598_2022_23993_MOESM1_ESM.doc]

# Highlights

- It is determined that the seed-filling stress is the largest when the inclination angle of metering device is 15 to 30 degrees
- The most significant factors affecting the seed-filling stress(*S*) were: inclination angle of the metering device (*θ*) > rotation speed of the metering disc (*n*) > machine ground speed (*v*).
- The most significant factors affecting the missed seeding rate(*M*) were: rotation speed of metering disc (*n*) > machine ground speed (*v*) > inclination angle of the metering device (*θ*).
- Only changed the inclination angle of seed-metering device cannot effectively reduce the missed seeding rate, and it needs to consider the rotation speed of metering disc and machine ground speed.
- The optimal combination of these operational parameters was *θ*=24^0^, *v*=2.73*m/s*, and *n*=10.5*rad/s*, providing a minimum *M*=5.25% and maximum *S* =0.77*Mpa*.
- Field verification test results indicated that the optimized corn seed-metering planter achieved mean values of *M*=4.33%, *Q*(qualified seeding rate) = 92.83% and *R*(repeated seeding rate)=2.84%.
